# National survey of therapeutic orientation and associated factors of counselors and psychotherapists in China

**DOI:** 10.3892/etm.2013.929

**Published:** 2013-01-28

**Authors:** XIAOMIN LIU, YUPING CAO, QIJIA SHI, CHANGQING JIANG, JIANXIN LIU, HONG WEI, XUEYAO MA, JIANQUN WAN, SHUYUN LV, LI HU, YING LIU, CHUNYING ZHOU, JUN ZHANG, YALIN ZHANG

**Affiliations:** 1 Mental Health Institute, Second Xiangya Hospital, Central South University, Changsha, Hunan;; 2 Psychiatric Department of the First Affiliated Hospital of Kunming Medical University, Kunming, Yunnan;; 3 Wuhan Hospital for Psychotherapy, Wuhan Mental Health Center, Huazhong University of Science and Technology, Wuhan, Hubei;; 4 Beijing Anding Hospital Affiliated to Capital University of Medical Science, Beijing;; 5 Mental Health and Life Education Center, Zhejiang University of Media and Communications, Hangzhou, Zhejiang;; 6 Tongji Hospital of Tongji Medical College, Huazhong University of Science and Technology, Wuhan, Hubei;; 7 Psychosomatic Department of the General Hospital of Ningxia Medical University, Yinchuan, Ningxia;; 8 Clinical Psychological Department of the Xinjiang Mental Health Center, Urumqi, Xinjiang;; 9 Psychiatric Department of the First Affiliated Hospital of China Medical University, Shenyang, Liaoning;; 10 College of Humanities and Social Sciences, Harbin Engineering University, Harbin, Heilongjiang;; 11 Mental Health Center, West China Hospital of Sichuan University, Chengdu, Sichuan, P.R. China

**Keywords:** therapeutic orientation, associated factors, counselors and psychotherapists, China

## Abstract

The aim of the present study was to determine the most commonly used and primary psychotherapeutic orientations adopted by Chinese practitioners and to examine the factors associated with the choice of orientation. A nationwide survey using multi-stage convenience sampling without replacement was conducted. A total of 1,232 respondents out of the 1,325 participants selected completed the survey, which corresponds to an overall response rate of 93.0%. The respondents were practitioners who were providing consultations and psychotherapy in China at the time. The main outcome measures were the most commonly used and primary psychotherapeutic orientations. A Chi-square test was used to examine the factors associated with therapeutic orientation. The most commonly used psychotherapies were cognitive therapy (59.2%), behavioral therapy (38.1%) and the psychoanalytic/psychodynamic model (29.4%). The primary orientations were cognitive therapy (41.6%), the psychoanalytic/psychodynamic model (15.7%) and cognitive-behavioral therapy (10.3%). Gender had no effect on the orientation choice. Cognitive therapy was used significantly more by respondents who were ≤30 years old (50.5%), who had been in practice ≤3 years (45.9%), received continuing education ≤64 h (47.2%) and accepted no clinical supervision (53.1%). Those who were ≥31 years old (18.4%), had been in practice ≥7 years (21.0%), received continuing education ≥65 h (23.6%), worked full-time (20.2%) and accepted clinical supervision (20.6%) used the psychoanalytic/psychodynamic model significantly more. The respondents who used cognitive-behavioral therapy had graduated from the medical profession (14.1%) and were not licensed (15.8%). Cognitive therapy and the psychoanalytic/psychodynamic model were the two most popular orientations adopted by Chinese counselors and psychotherapists. Age, years of practice, graduate profession, continuing education, working hours (full/part-time), licensure and supervision are significant factors that affect the choice of orientation.

## Introduction

Since the Chinese Ministry of Labour and Social Security held the first qualifying examination for professional counselors in 2003 (1), counseling and psychotherapy have received a great deal of attention. The number of practitioners working in general and psychiatric hospitals was ∼12,000 in 2007 (2), which was an increase of 12% from 2001 (3).

As the therapeutic orientation that counselors and psychotherapists adopt has implications for the process and outcome of treatment, a number of Western psychologists have been exploring this factor since the 1970s. These studies suggested that numerous factors were associated with the choice of orientation, including age (4,5), supervision (5), graduate profession (5), training (4,6,7) and clinical experience (6). Almost every type of psychological theory and technique has its pupils and practitioners in China, but there have been few studies on the therapeutic orientation of Chinese counselors and psychotherapists and no studies on the associated factors.

The settings for Chinese psychological employment are divided into three disciplines, specifically, the healthcare system, the educational system and that of other practitioners. In a survey of clinical psychologists in 1996, the most commonly used psychotherapies were behavioral therapy, cognitive therapy, cognitive-behavioral therapy (CBT), supportive psychotherapy, psychoanalysis, Morita’s therapy and client-centered therapy (8). In another study completed in 2009, based on the psychological practitioners in middle and primary schools, the most common orientations were client-centered therapy, followed by cognitive therapy, psychoanalysis, family therapy and Morita’s therapy (9). In 2008, a study of 15 private psychological settings observed the use of integrative orientation, followed by CBT, psychoanalysis, family and humanistic therapies (10). However, all these studies had certain limitations, for example, they restricted the number of psychotherapies that could be chosen, were limited to certain regions or work settings and did not analyze the factors affecting the choice of therapeutic orientation. We therefore conducted a national wide survey on the therapeutic orientation of counselors and psychotherapists, considering the affects of socioeconomic level and employment setting and their associated factors.

## Subjects and methods

### Development of the survey

The survey was one of the core research elements of the ‘Standardizing and modeling ten kinds of psycho-counseling and psychotherapy in China’ project. The goal of the project which provided support to the survey was to standardize the operation and formulate the guide for ten assigned psychotherapies, therefore the main purpose of the survey was to study the psychotherapies adopted by the Chinese counselors and psychotherapists, and how they practice. We referred former studies to formulate the questionnaire and referred to the suggestions of a few senior psychotherapists who provided their opinions on how to study the psychotherapies. A draft questionnaire titled the ‘National Status Quo Survey on Counselors and Psychotherapists in China’ was developed. In total, 2 pilot studies were conducted; the questionnaire was modified twice according to the pilot study and revised for the third time according to the views of the experts who joined the first conference of the project. The final questionnaire consisted of 44 questions, divided into 6 sections on demography, working modes, techniques and efficiency, learning and training, clients, and expectations and other. The survey took 15–20 min to complete. All participants provided written informed consent and the present study was approved by the Ethics Committees of the Second Xiangya Hospital and Central South University (Changsha, China).

### Sampling

Due to a lack of credible statistics on the consultations and psychotherapy practitioners in China, multi-stage convenience sampling without replacement was conducted. The sampling procedure was divided into three steps. Firstly, according to the administrative divisions of China defined by the government, the Chinese mainland was divided into 6 regions; the northeast, north, central south, east, northwest and southwest of China, termed as regions 1 to 6, respectively. The sampling cities were taken from all 6 regions. Secondly, within each region, the sampling cities were divided into 3 city levels; A for the well-developed, B for the moderately developed and C for the underdeveloped, based on their socioeconomic development. In the survey, the socioeconomic development of the sampled city was represented by the quantities and qualities of the treatments that the practitioners offered. Thirdly, the sampling disciplines included three groups; the healthcare system, educational system and other practitioners, including those from private sectors, enterprises and prisons. Although the populations of the regions varied greatly, there were hundreds of practitioners working in each region. A quota sample of 200 practitioners was selected from each region, with a total of 1,200 samples selected. It was observed that the number of practitioners who worked in level-A cities was much larger than that in level-B or C cities and that overall there were extremely few practitioners working in level-C cities. According to the possible number of practitioners based in each level of city, level-A represented 60% of the sampling, with levels B and C representing 30 and 10%, respectively. Every discipline was represented by one-third of the samples in each region and city-level.

The subjects of the sampling were the practitioners who were providing consultations and psychotherapy at the time. As numerous counselors and psychotherapists working in China had no qualification certificate, whether they owned a qualification certificate or not was not considered. There were three methods for locating potential participants. Firstly, the researchers began the survey on practitioners that they had previously met, then the information on other practitioners was obtained through an introduction by these known practitioners. Secondly, the researchers conducted a retrieval of data from the internet using certain key words, including psychotherapist, psychologist and psychological studio, then they searched for the potential practitioners who had a definite means of contact. Thirdly, the researchers obtained a list of trainers from various psychological training institutions and selected those who performed psychological consultations and psychotherapy as potential practitioners.

The survey was conducted as a face-to-face interview as often as possible, however, hard copies of the questionnaire were mailed out or delivered to the person in charge of the psychological setting and then collected if the respondent preferred. The present study did not conduct any research outside of China. The survey began in October 2010 and ended in November 2011 subsequent to each region completing >200 sample responses.

### Data collection and analysis

The respondents were asked to write down their most commonly used psychotherapies according to their order of frequency on a blank form. The treatment setting and a subjective evaluation of the efficiency of the psychotherapy were requested to ensure that the psychotherapies noted were those that were most commonly used and not those that the respondent was most interested in. The number of psychotherapies each respondent was able to include was not limited. As the psychotherapies included were not repeated, the frequencies were calculated and the top 10 were labeled as the ‘Top 10 most commonly used psychotherapeutic orientations’ (Table I). The psychotherapy that the respondent noted first was considered to be the primary orientation; the top 6 were named as the ‘Top 6 primary psychotherapeutic orientations’ (Table II).

In total, 9 factors associated with the therapeutic orientation were selected for examination: gender, age, years of practice, graduate profession (psychology, medicine, education and others), method of continuing education (conference, advanced study, training course, clinical supervision, self-study and web-based study), hours of training received in the last year, working hours (full/part-time), licensure (yes/no) and acceptance of supervision (yes/no).

Of these factors, the age, years of practice and hours of training received were continuous variables as their raw data and transforming data did not fit the conditions of a parametric test. A nonparametric test (Kruskal-Wallis H test) was conducted, then the factors were re-classified into three subgroups in order to make comparisons. The groups for: age were ≤30, 31–39 and ≥40 years; for years of practice were ≤3, 4–6 and ≥7 years; and for hours of training received were ≤25, 26–64 and ≥65 h.

### Statistical analysis

SPSS 19.0 statistical software was employed for data entry and analyses. A Chi-square test was used to examine the differences within the categorical variables. The significance level was α=0.05 (2-tailed) and a Bonferroni correction was used for each family of pairwise tests. If there were 3 categories, each family of tests involved 3 comparisons and α was set at 0.0167 (=0.05÷3) for each comparison. If there were 4 categories, α was set at 0.0083 (=0.05÷6). For 6 categories, α was set at 0.0033 (=0.05÷15).

## Results

### Response rate

Of the 1,325 subjects, 44.5% (n=590) completed the questionnaire through face-to-face interviews and the response rate for this method was 100%. The other 55.5% (n=735) of the participants were sent questionnaires by post or by courier and the response rate was 87.3% (n=642). The overall response rate was 93.0% (n=1,232). The item-response rate for the ‘most commonly used psychotherapeutic orientations’ was 96.5% (n=1,189) and for the 9 associated factors the rate varied between 90.0 and 99.8%.

### Participant proportion

In total, 1,232 participants completed the survey (regions 1 to 5 had 200 respondents each, while region 6 had 232). Cities of levels A, B and C had 733 (59.5%), 367 (29.8%) and 132 (10.7%) responses respectively. A total of 419 participants (34.0%) belonged to the educational system, 391 (31.7%) came from healthcare system and 399 (32.4%) were other practitioners.

### Top 10 most commonly used psychotherapeutic orientations

A total of 63 psychotherapies were selected 2,937 times. Table I shows the 10 most commonly used orientations. In total, 70.1% (n=839) of the responses had ≥2 common orientations and 5.7% (n=68) used integrative therapy as their most commonly used psychotherapy. Within the group that selected integrative therapy, CBT was mentioned as the core theory by 26.4% (n=18), followed by cognitive orientation which was mentioned by 18.9% (n=13), and psychoanalysis and humanistic therapy which both accounted for 17.0% (n=12).

### Top 6 primary psychotherapeutic orientations

A total of 33 orientations were indicated 1,189 times by the responses. Table II shows the 6 primary psychotherapies. Although there was a difference between the most commonly used and primary psychotherapy orientations, cognitive therapy was ranked first for each of the results. For 59.2% (n=704) of the respondents, cognitive therapy was one of the most commonly used psychotherapies and for 41.9% (n=498) it was the primary psychotherapeutic orientation.

### Factors associated with the primary therapeutic orientations. Gender

In total, 70.2% (n=778) of the participants were female. When the primary therapeutic orientations were analyzed by gender, there was no significant difference between the female and male practitioners.

### Age

The age of the practitioners sampled ranged from 21 to 80 years, the median age was 36 years and the mean age was 37.01 years (SD=8.428), with half of the respondents (50.9%) <36 years old. The practitioners that adopted the various primary psychotherapies had significantly different mean ages (χ^2^=31.919, P<0.001). The respondents who selected behavioral therapy were the youngest with a mean age of 34.96 years (SD=6.441) and those who selected integrative orientation were the oldest with a mean age of 39.69 years (SD=8.385).

Table III summarizes the primary psychotherapies of the survey respondents in the three age groups. Cognitive therapy was adopted by the highest proportion of practitioners in all three age groups, but was observed at a significantly higher rate in the ≤30 years group than in the other two groups (χ^2^=9.570 and 9.260; P<0.01). CBT and psychoanalytic/psychodynamic psychotherapy accounted for 11.5 and 7.2% of responses, respectively, in the ≤30 years group. These results were is the reverse order in the 31–39 and ≥40 years groups with a higher proportion of respondents selecting psychoanalytic/psychodynamic psychotherapy (19.1 and 17.5%, respectively) than CBT (9.8 and 10.5%, respectively).

### Years of practice

The participants had been in practice an average of 5.82 years (SD=4.812), the length of practice ranged from <1 year to 43 years and two-thirds (60.3%) had been in practice for ≤5 years. The respondents who selected behavioral therapy had been in practice the shortest time with a mean of 4.79 years (SD=3.547) and those who selected the psychoanalytic/psychodynamic model had been in practice the longest time with a mean of 6.71 years (SD=4.691). The mean number of years of practice was significantly different in the responses of those who employed varying primary psychotherapies (χ^2^=29.767, P=0.001).

Table III shows the primary orientation that practitioners adopted in the three groups for years in practice. There was a significant correlation between the years of clinical experience and the use of the cognitive or psychoanalytic orientations. The results showed that novice (≤3 years) practitioners used the cognitive therapy significantly more than senior practitioners (≥7 years; χ^2^=6.008, P<0.0167) and that the senior practitioners used psychoanalysis significantly more than the novices (χ^2^=13.750, P<0.001).

### Graduate profession

In total, 14.1% of the responders from those in the medical profession selected CBT as their primary orientation. This proportion was higher than that of the psychological or educational professions (11.0 and 9.2%, respectively) and significantly higher than that of ‘other’ professions (5.6%; χ^2^=12.936, P<0.001). When analyzed by graduate profession, no significant differences were observed in the other five primary psychotherapies.

### Method of continuing education

With regards to the method of continuing education, the results showed that the respondents who were continuing their education via conference (41.1%; χ^2^=13.391, P<0.001), advanced study (40.2%; χ^2^=12.430, P<0.001), training course (40.7%; χ^2^=16.121, P<0.001) and self-study (42.2%; χ^2^=19.979, P<0.001) used cognitive therapy significantly more than those continuing via clinical supervision (30.5%). The opposite was true for those who indicated a use of the psychoanalytic/psychodynamic model. The proportion of participants who were educated via clinical supervision (25.0%) used psychoanalysis significantly more than those using conferences (16.0%, χ^2^=9.337, P<0.0033), advanced study (16.8%, χ^2^=14.964, P<0.001), training courses (17.4%, χ^2^=15.007, P<0.001) and self-study (15.9%, χ^2^=18.106, P<0.001).

### Hours of training

The hours of training received in the last year encompassed a large scope of hours ranging between 0 and 1,200 h. The mean number of hours was 69.44 (SD=94.907), with one-third of the respondents (31.9%) undertaking <20 h of training. The respondents who trained for the fewest hours, with a mean of 47.16 h (SD=40.786), made use of client-centered/humanistic therapy, while those who trained the longest, with a mean of 85.61 h (SD=77.233), selected the psychoanalytic/psychodynamic model. The mean number of hours were significantly different when the primary psychotherapies were analyzed by the hours of training (χ^2^=64.955, P<0.001).

The practitioners were grouped into those with low (0–25 h), medium (26–64 h) and high (>65 h) training hours. The practitioners with low (49.2%, χ^2^=28.703, P<0.001) and medium training hours (45.2%, χ^2^=17.837, P<0.001) used cognitive therapy significantly more and the psychoanalytic/psychodynamic model significantly less than the practitioners with high training hours (9.9, 15.2 and 23.6%, respectively; χ^2^=24.727 and 7.424; P<0.001). The practitioners with medium training hours (3.3%) made use of behavioral therapy significantly less than the practitioners with low (8.6%; χ^2^=9.214, P=0.002) and high training hours (7.4%; χ^2^=5.829, P<0.0167).

### Full/part-time, licensure and acceptance of supervision

The participants included 47.2% (n=580) who worked full-time, 76.8% (n=938) who were licensed and 64.1% (n=776) who were supervised. Table IV presents information on the primary psychotherapies analyzed by working hours (full/part-time), licensure (yes/no) and acceptance of supervision (yes/no). The factors of working full-time and accepting supervision had a significant effect on the use of psychoanalysis, no licensure had a significant effect on the use of CBT and accepting no supervision was significantly correlated with the use of cognitive therapy.

## Discussion

In certain surveys conducted in America (11), Australia (12) and Portugal (6), cognitive therapy was the most prevalent psychotherapeutic orientation, followed by the psychoanalytic/psychodynamic model. The present survey draws the same conclusions as more than half of the respondents indicated one of these as their primary orientation. The findings also showed that despite the emergence of numerous new types of psychotherapies, the ‘classical’ psychotherapeutic orientations are more common in China. From observing the results, we were disappointed to see that neither the top 10 most commonly used psychotherapies nor the top 6 primary psychotherapies contained traditional Chinese psychotherapies such as Taoist cognitive psychotherapy and Qigong treatment.

Published studies reflect the status quo of counseling and psychotherapy. A review (13) of studies published in Chinese psychological journals between 2000 and 2009 showed that the integrative theory was introduced in the majority of the journals (37.18%), followed by the cognitive (17.66%) and behavioral theories (8.62%). The majority of psychological theories were adopted from the West (93.7%), so the indigenized and indigenous theories accounted for extremely small proportions (4.41% and 1.26%, respectively). From the results of this review, we may assume that integration was introduced most commonly as a response to psychological tendency. The fact that the introduction of the cognitive and behavioral theories ranked second and third, respectively, may partly explain why the two psychotherapies are the two most popular orientations commonly used by Chinese practitioners. From this previous study it is also possible to infer that as the psychological service is a new discipline and a new occupation that came from the West to China, the majority of theories that are widely spread and learned are foreign, as are the therapeutic orientations adopted. The traditional views on mental health, including‘letting things slide’ and ‘abstinent contentment’ have been engrained in Chinese daily life for thousands of years, however, they have only been termed as ‘psychology’ or ‘counseling and psychotherapy’ for decades. As a result, it may be a long time until these views are framed into theories which are widely spread and learnt. This would partly explain the reasons that the indigenous therapies are neither the commonly used nor primary psychotherapies.

Integration may be considered as a sign of academic maturation and this is the case for psychotherapy. Certain researchers believe that integration is an emerging and continuing trend in psychotherapy (14). Integrative orientation accounted for 15% of the responses in the American Psychologists Association (APA) survey held in 2008 (11), while in a study of Portuguese therapists this proportion was 13% (6). In the present study, 5.7% of the respondents indicated that they used integrative therapy; the low proportion suggests that the psychological service is a ‘young’ profession in China. The practitioners making use of integrative therapy were the more senior respondents, which is also an endorsement for this trend.

Integration should be based on the most commonly used psychotherapies as practitioners are not able to integrate the orientations that they do not know well. In accordance with this view, in a study in Argentina, psychoanalysis was chosen by 53.1% of psychotherapists as their primary orientation and was mentioned by 63.2% of the integrationists as the primary theory (15). In the present survey, CBT, cognitive therapy, psychoanalytic therapy and humanistic orientation were chosen as the base theory by nearly one-fifth of the sample. These were all also commonly used psychotherapies and primary orientations.

In numerous studies of therapeutic orientation, cognitive therapy and the psychoanalytic/psychodynamic model appeared to be the two ‘extremes’ which were usually selected for comparison with each other. The present study supports this view. There were significant differences between the two orientations when almost all the factors affecting the primary psychotherapies were analyzed.

In a number of surveys, females have been observed to be working in the psychological services in greater numbers than males. For example, a previous study in Australia observed that 74.6% of practitioners were female (16), while in a similar study conducted in China (2), females represented 59.0%. In the present study, 70.2% of the sample was female. There was no significant difference between the female and male practitioners in terms of their primary therapeutic orientations.

Compared with practitioners in certain foreign studies, Chinese counselors and psychologists are younger and have been in practice for a shorter time. For instance, in a previous study of APA members the mean age was 53 years with nearly half >55 years. The participants were licensed for an average of 17 years and only one-fifth were licensed for <5 years (11). In the present study the mean age was 37.01 years with half <36 years. The mean time in practice was 5.80 years with three-fifths <5 years. This situation also reflects the fact that the psychological service is ‘young’ in China.

When considering age as a factor, the relatively consistent conclusions drawn from past studies have been that older psychologists tended to follow the psychoanalytic/psycho-dynamic model (4,5) and that the younger psychologists were more likely to be eclectic/integrative or follow newer approaches (5), CBT (4) or behavioral orientation (17). The present study obtained certain similar results and also some new findings. The older respondents were prone to using the psychoanalytic/psychodynamic model while the younger respondents were prone to using cognitive and behavioral therapy, however the respondents who selected integrative orientation tended to be the older respondents.

The length of practice increases in parallel with age. The participants who are <30 years old and have been in practice <3 years used cognitive therapy significantly more than the respondents who were >31 years old and had been in practice >7 years. By contrast, the latter category used the psychoanalytic/psychodynamic model significantly more than the former. This may imply that novices tend to use cognitive therapy and that experienced psychologists tend to use the psychoanalytic/psychodynamic model.

In this survey, all the associated factors expect gender and graduate profession had a significant effect on cognitive therapy and psychoanalytic/psychodynamic orientation, and graduate profession had no significant effect on the two orientations but had a significant effect on use of the CBT. In the present study, this had no significant effect on the use of the psychoanalytic/psychodynamic orientation but had a significant effect on the use of CBT. These findings support the view that graduate psychological training is one of the most significant determinants for the choice of CBT, but not for psychoanalysis (4). In China, medical graduates usually work in healthcare settings, including hospitals, hence, in accordance with past studies (18,19), the findings of the present study revealed that clinical psychology practitioners were prone to using CBT. A plausible explanation for this observation is that the healthcare discipline focuses on the treatment of mental disorders and that the initial models of CBT were developed to address mental disorders, including anxiety and depression (19).

Opportunities for training have a significant effect and cause restrictions in the psychotherapists’ choice of orientation (7). The preference for psychodynamic therapy is affected to a large extent by training factors (20). Consistent with previous studies, the present study demonstrated that the method and hours of training have a significant effect on the choice of cognitive and psychodynamic orientation. Particularly, the psychoanalytic/psychodynamic model was significantly associated with long training hours and training involving clinical supervision.

In the present survey, 47.2% of respondents were working full-time. This result was close to the proportion of 40.1% observed in a similar study previously carried out in China (2) and is lower than the result of 67.7% observed in the APA survey (2008)(11). Full-time practitioners used the psychoanalytic/psychodynamic model significantly more than part-time workers. This may be due to full-time practitioners spending more time in training and having more opportunities to obtain clinical supervision.

The findings from the APA survey (2008) showed that 95% of practitioners were licensed and that the remaining 5% included psychologists working in settings not requiring professional licensure (11). The present survey revealed that 76.8% of the Chinese participants were licensed. This proportion was lower than that in the APA survey. Currently, professional licensure is not a necessary requirement for psychological practice in China. As an estimate, only one-third of licensed practitioners work in psychological settings (3), while a number of psychological practitioners have no professional licensure. In the present study the respondents who had no licensure selected CBT as their primary psychotherapy significantly more than their licensed counterparts. In China, practitioners with a medical background usually own a license to practice medicine rather than a certificate for psychology, hence the proportion of counselors and psychotherapists with a medical background who have no licensure is relatively high. The practitioners in the medical graduate profession were those who principally selected CBT; this may be one of the reasons for the correlation between a lack of licensure and the use of CBT.

In a Chinese survey held in 2009 (21), 58% of the practitioners accepted clinical supervision. The proportion of such practitioners was 64.1% in the present sample, which is higher than that in the previous study. This increase enforces the importance of supervision being recognized by practitioners and reflects the development of the professionalization of the psychological services in China. Accepting clinical supervision has a significant effect on the use of the psychoanalytic/psycho-dynamic model and no supervision is significantly associated with the use of cognitive therapy. ‘Supervision’ began informally in 1902 when it was incorporated into Wednesday night meetings at Freud’s home (22). From this, it may be observed that supervision and psychoanalysis/psychodynamics have a shared origin. At the same time, supervision is a required process for psychoanalytic training. These reasons may explain the significant correlation observed between supervision and the psychoanalytic/psychodynamic model.

In summary, the present study suggests that cognitive therapy is the most prevalent psychotherapy in China, followed by the psychoanalytic/psychodynamic model and CBT. The novices, i.e., those receiving shorter training hours and accepting no clinical supervision, have been identified as using cognitive therapy significantly more. The experienced, i.e., those who receive longer training hours, work full-time and accept clinical supervision, tend to follow the psychoanalytic/psychodynamic model. The respondents who select CBT significantly more are those who have graduated from a medical profession and are not licensed.

The present study has several limitations. Firstly, it does not distinguish between the initial therapeutic orientation and the current orientation, so there is no differentiation between whether the practitioners have changed orientations during their clinical experiences or not. Further studies are therefore required to investigate the initial therapeutic orientation and the change in orientation in Chinese counselors and psychotherapists, as well as the factors associated with these selections. Secondly, numerous researchers believe that factors, including training, supervision and clinical experiences, may determine the initial choice of orientation, but that personality traits and epistemological values are more significant determinants in maintaining a therapeutic orientation (23,24). Consequently, studies into personality traits and epistemological values and their affect on the choice of orientation in Chinese practitioners are also required.

## Figures and Tables

**Table I:** Top 10 most commonly used psychotherapeutic orientations.

	Responses (n=2937)		
		
Orientation	N	Percentage	Percentage of cases (n=1189)
Cognitive therapy	704	24.0	59.2
Behavioral therapy	453	15.4	38.1
Psychoanalytic/psychodynamic psychotherapy	349	11.9	29.4
Family therapy	190	6.5	16.0
Cognitive-behavioral therapy (CBT)	186	6.3	15.6
Client-centered/humanistic therapy	185	6.3	15.6
Sandplay therapy	112	3.8	9.4
Hypnotherapy	102	3.5	8.6
Integrative therapy	68	2.3	5.7
Morita’s therapy	66	2.2	5.6
Other (53 orientations were listed)	522	17.8	43.9

**Table II:** Top 6 primary psychotherapeutic orientations.

Orientation	Responses (n=1189)	Percentage of cases
Cognitive therapy	498	41.9
Psychoanalytic/psychodynamic psychotherapy	187	15.7
Cognitive-behavioral therapy (CBT)	123	10.3
Behavioral therapy	76	6.4
Client-centered/humanistic therapy	48	4.0
Integrative therapy	45	3.8
Other (27 orientations were listed)	212	17.9

**Table III:** Primary therapeutic orientation that practitioners adopted in the varying groups for age and number of years in practice.

	Age group (years)			Years of practice		
Orientation	≤30 [a] (n=305)	31–39 [b] (n=491)	≥40 [c] (n=371)	≤3 [d] (n=438)	4–6 [e] (n=365)	≥7 [f] (n=367)
Cognitive therapy	50.5^bc^	39.4	38.8	45.9^f^	42.5	37.3
Psychoanalytic/psychodynamic psychotherapy	7.2	19.2^a^	17.5^a^	11.4	16.2	21.0^d^
Cognitive-behavioral therapy (CBT)	11.5	9.9	10.5	10.3	10.4	10.1
Behavioral therapy	6.2	8.2^c^	3.8	7.5	4.7	5.7
Client-centered/humanistic therapy	5.9	3.4	3.8	5.0	3.0	3.8
Integrative therapy	3.0	2.5	6.5^b^	2.5	5.5	3.5
Other (27 orientations were listed)	15.7	17.4	19.1	17.4	17.7	18.6

The letter in [ ] is assigned to represent the column variables. For each significant pair, the letter of the smaller category is placed over the category with the larger proportion. Since there are 3 levels of age groups and years of practice, 3 pairs of columns are compared in each set of tests and Bonferroni adjustments are used to the adjust significance level. α was set at 0.0167 (=0.05÷3). All values are percentages.

**Table IV:** The primary therapeutic orientation adopted by practitioners analyzed by working hours (full/part-time), licensure and acceptance of supervision.

	Working hours		Licensure		Supervision	
			
Orientation	Full-time [a] (n=570)	Part-time [b] (n=617)	No [c] (n=273)	Yes [d] (n=908)	No [e] (n=617)	Yes [f] (n=617)
Cognitive therapy	39.8	43.9	42.5	41.5	53.1^f^	35.7
Psychoanalytic/psychodynamic psychotherapy	20.2^b^	11.7	13.2	16.5	6.7	20.6^e^
Cognitive-behavioral therapy (CBT)	10.0	10.7	15.8^d^	8.8	11.0	10.3
Behavioral therapy	5.4	7.3	6.6	6.2	6.2	6.4
Client-centered/humanistic therapy	3.9	4.1	2.6	4.5	3.1	4.3
Integrative therapy	3.3	4.2	3.3	4.0	3.8	3.9
Other (27 orientations were listed)	17.4	18.1	16.0	18.5	16.1	18.8

The letter in [ ] is assigned to represent the column variables. For each significant pair, the letter of the smaller category is placed over the category with the larger proportion. The significance level was α=0.05 (2-tailed). All values are percentages.
